# Evaluation of a 27-gene inherited cancer panel across 630 consecutive patients referred for testing in a clinical diagnostic laboratory

**DOI:** 10.1186/s13053-017-0083-8

**Published:** 2018-01-04

**Authors:** Sabrina A. Gardner, Katelyn S. Weymouth, Wei S. Kelly, Ekaterina Bogdanova, Wenjie Chen, Daniel Lupu, Joshua Suhl, Qiandong Zeng, Ute Geigenmüller, Debbie Boles, Patricia M. Okamoto, Geraldine McDowell, Melissa A. Hayden, Narasimhan Nagan

**Affiliations:** Integrated Genetics, Laboratory Corporation of America® Holdings, Research Triangle Park, NC and 3400 Computer Drive, Westborough, MA 01581 USA

**Keywords:** Inherited cancer panel, VUS, HBOC, Lynch syndrome, CMMRD, Variant classification

## Abstract

**Background:**

Extensive clinical and genetic heterogeneity of inherited cancers has allowed multi-gene panel testing to become an efficient means for identification of patients with an inherited predisposition to a broad spectrum of syndromic and nonsyndromic forms of cancer. This study reports our experience with a 27-gene inherited cancer panel on a cohort of 630 consecutive individuals referred for testing at our laboratory with the following objectives: 1. Determine the rates for positive cases and those with variants of uncertain clinical significance (VUS) relative to data published in the recent literature, 2. Examine heterogeneity among the constituent genes on the panel, and 3. Review test uptake in the cohort relative to other reports describing outcomes for expanded panel testing.

**Methods:**

Clinical and genomic data were reviewed on 630 individuals tested on a panel of 27 genes selected on the basis of high (≥ 40%) or moderate to low (≤ 40%) lifetime risk of hereditary cancer. These patients were not enriched for adherence to the National Comprehensive Cancer Network (NCCN) criteria for Hereditary Breast and Ovarian Cancer (HBOC) or Lynch Syndrome (LS) and constitute a referral laboratory cohort.

**Results:**

Sixty-five individuals with variants classified as pathogenic or likely pathogenic across 14 genes were identified for an overall positive rate of 10.3%. Although a family history of cancer constituted a major reason for referral, accounting for 84% of our cohort, excluding patients with a known familial variant did not have a significant impact on the observed positive rate (9% vs 10.3%). More than half (58%) of the pathogenic or likely pathogenic variants were observed in high or moderate to low risk genes on the panel, while only 42% occurred in classic HBOC or LS-associated genes.

**Conclusion:**

These results provide the actual percentage of family or personal history of cancer that can be attributed to pathogenic or likely pathogenic variants in one or more of the genes on our panel and corroborate the utility of multi-gene panels over sequential testing to identify individuals with an inherited predisposition to cancer.

**Electronic supplementary material:**

The online version of this article (doi: 10.1186/s13053-017-0083-8) contains supplementary material, which is available to authorized users.

## Background

Hereditary Cancer Syndromes, including Hereditary Breast and Ovarian Cancer (HBOC) and Lynch Syndrome (LS), can result in various forms of cancer due to germline mutations in cancer predisposition genes. While the major contributory genes for these syndromes have been identified and well-studied (*BRCA1*/*BRCA2* for HBOC and *MSH2*/*MSH6*/*MLH1*/*PMS2/EPCAM* for LS), there remains a large percentage of associated cancer cases that are negative for germline mutations in these genes, including 80% of women with a personal or family history of breast cancer who are negative for *BRCA1/2* mutations [1]. Similarly, between 30 and 50% of families fulfill stringent criteria for LS and test negative for germline mismatch repair gene mutations [2]. Adding complexity to these disorders is the significant overlap in the spectrum of cancers observed between various hereditary cancer syndromes, including many cancer susceptibility syndromes. Some that contribute to elevated breast cancer risk include Li-Fraumeni Syndrome, Cowden syndrome, Hereditary Diffuse Gastric Cancer, Ataxia-telangiectasia, Lynch Syndrome, and Peutz-Jeghers syndrome [3], while others that contribute to elevated colorectal cancer risk include Familial Adenomatous Polyposis and *MUTYH*-associated Polyposis [4]. Additionally, the risk of cancers observed within these syndromes can vary widely. HBOC not only increases the life-time risk of breast and ovarian cancers, but also for prostate and pancreatic cancers [3]. Similarly, LS-spectrum cancers include colorectal cancer, as well as uterine (endometrial), ovarian, gastric, and other rare forms of cancer [5].

Over the past decade, linkage, GWAS, and re-sequencing studies have identified additional genes that contribute to the risk of hereditary cancer [6]. The discoveries of these genes, which vary in their level of penetrance and risk-contribution, present opportunities for personalized clinical management in patients and families with hereditary cancer.

Over time, multi-gene panels for hereditary cancer continue to grow in complexity, from sequencing only the major contributory genes for HBOC and LS, a total of roughly six genes, now expanding to 27 genes and higher [2, 7–14]. The addition of less characterized genes to hereditary cancer panels poses challenges in interpretation due to lack of available information such as disease prevalence, penetrance, locus/allelic heterogeneity, expressivity, age of onset, and the spectrum of gene-associated cancers. Three recent studies reporting the clinical outcomes of panel-based testing for inherited cancer have utilized cohorts selected for patients fulfilling the National Comprehensive Cancer Network (NCCN) guidelines for breast cancer [7] or colorectal cancer [2], or Society of Gynecologic Oncology 5–10% criteria for endometrial cancer [9]. One of the studies used retrospective history-enriched cases from clinical bio banks with referrals based on known familial pathogenic variants [7]. In this study, we report our experience with a 27-gene inherited cancer panel on a cohort of 630 consecutive patients referred for testing at our laboratory. Our objectives were: 1. Determine the rates for positive cases and those with variants of uncertain clinical significance (VUS) relative to data published in the recent literature, 2. Examine heterogeneity among the constituent genes on the panel, and 3. Review test uptake in the cohort relative to other reports describing outcomes for expanded panel testing.

## Methods

### Participants

Clinical and genomic data were reviewed for the first 630 consecutive individuals referred for hereditary cancer gene panel testing at our clinical laboratory improvement amendments (CLIA)-approved commercial laboratory (Integrated Genetics, Laboratory Corporation of America® Holdings, Research Triangle Park, NC). Relevant information including the clinical indication for testing, family history, and demographic data were obtained by review of the ordering test requisition forms. For clinical diagnostic testing, it is standard for the referring physician to obtain informed consent prior to test ordering; therefore, an ethics approval was not required. Individuals referred for testing were not restricted to any specific center or region and spanned a broad referral pattern. Patients were stratified based on personal and family history of HBOC-associated cancers (breast, ovarian, prostate) as well as LS-associated cancers (colorectal, gastric, pancreatic, ovarian, uterine/endometrial, and bladder). As ovarian cancer is a common clinical feature of both HBOC and LS, patients with ovarian cancer were included in both these categories.

### Gene panel VistaSeq^SM^ hereditary cancer panel

Genomic DNA was isolated from blood specimens using Chemagic DNA Blood Kit Special (PerkinElmer, Shelton, CT). DNA was quantified on a NanoDrop spectrophotometer (ThermoFisher Scientific, Charlotte, NC), normalized, and fragmented on a Covaris ultrasonicator instrument (Woburn, MA). Target enrichment for the entire gene coding regions, as well as all flanking non-coding regions, of 27 cancer genes (Table 1) was performed using custom SureSelectXT targeted capture probes (Agilent, Santa Clara, CA).

**Table 1:** List of 27 Genes analyzed by the Inherited Cancer Panel

Gene Set	Gene
BRCA	*BRCA1* (NM_007294.2), *BRCA2* (NM_000059.3)
Lynch Syndrome	*MLH1* (NM_000249.2)*, MSH2* (NM_000251.1)*, MSH6* (NM_000179.2)*, PMS2* (NM_000535.4)*, EPCAM* (NM_002354.2)^a^
High Risk for Breast and other Hereditary Cancer Syndromes	*APC* (NM_000038.3)*, BMPR1A* (NM_004329.2), *CDH1* (NM_004360.2), *CDK4* (NM_000075.2), *CDKN2A* (NM_000077.3), *MUTYH* (NM_001128425.1)^b^*, PTEN* (NM_000314.4), *SMAD4* (NM_005359.3), *STK11* (NM_000455.4), *TP53* (NM_000546.3)
Moderate to Low Risk Genes	*ATM* (NM_000051.3)*, BARD1* (NM_000465.2)*, BRIP1* (NM_032043.1), *CHEK2* (NM_007194.3), *FAM175A* (NM_139076.2), *NBN* (NM_002485.4), *PALB2* (NM_024675.3), *PRKAR1A* (NM_002734.3), *RAD51C* (NM_058216.1), *RAD51D* (NM_002878.2)

^a^MLPA only

^b^*MUTYH*-associated polyposis are caused by biallelic mutations in *MUTYH*

The enriched libraries were clonally amplified on a solid substrate for NGS using the Illumina V3 chemistry on a MiSeq System (Illumina, San Diego, CA) to an average coverage of 300X with 2 × 150 paired-reads and a minimum coverage of 15X (with a variant allele frequency cutoff of 20.0%) for each targeted position, except for *PMS2* exons 12 and15 (which share a high degree of sequence homology to the *PMS2CL* pseudogene [15]), where a minimum coverage of 25X (with a variant allele frequency cutoff of 8.0%) was applied at each targeted position. Reads were aligned to the hg19/GRCh37 reference human genome build using an internally validated analysis workflow on the CLCBio™ platform (QIAGEN Bioinformatics, Redwood City, CA). A minimum quality threshold of Q20 that translates to a sequencing accuracy of >99% for all called bases was applied. Copy number variations were assessed by a custom microarray comparative genomic hybridization chip (Agilent Technologies, Santa Clara, CA) or multiple-ligation probe amplification (MRC Holland, Amsterdam, The Netherlands) analysis to detect gene deletions and duplications. All reportable sequence variants were confirmed by Sanger sequencing. All low coverage target regions were rescued by targeted amplification and Sanger sequencing, and reportable *PMS2* variants were confirmed by long-range PCR to exclude the *PMS2CL* pseudogene as their source. The technical and performance characteristics for all components were internally validated following guidelines set forth by the College of American Pathologists (CAP) [16].

### Variant classification

Variants were classified by a tiered in-house variant classification protocol in accordance with guidelines issued by the American College of Medical Genetics and Genomics [17]. Our variant classification is an algorithmically-weighted assessment that incorporates the following elements: 1. Prevalence of disease phenotypes associated with the constituent genes on the panel, 2. Pre-curated knowledge of gene and disease-specific properties, including allelic and locus heterogeneity, 3. Frequency of the variant in an unaffected (general) population as ascertained from the Exome Aggregation Consortium (ExAC) [18], Exome Variant Server [19], dbSNP, 1000 Genomes [20], and available literature, 4. Evidence of co-segregation in affected individuals, 5. Critical review of publically available content in variant databases (including but not limited to HGMD, ClinVar, OMIM, Breast Cancer Information Core (BIC), BRCA Share™ [21] and InSiGHT [22]), 6. Observed/reported co-occurrence with other deleterious variants within and between the genes on the panel as ascertained from external and internal testing databases, 7. Published evidence in controlled experimental systems linking a variant to established mechanisms of disease, and 8. Functional impact predictions using a variety of *in-silico* prediction tools. Pathogenicity of variants located in canonical splice sites and/or exon-intron boundaries was assessed using 5 splice prediction tools integrated into the ALAMUT™ software (Interactive Biosoftware, Rouen, France). Positive and VUS variants identified in this study will be submitted to the ClinVar database.

### Data analysis

The 27-gene panel was divided into four gene sets based on disease-association and level of risk/penetrance. These included genes involved in HBOC, LS, high risk (≥40%) and moderate to low risk (≤40%) genes for other forms of hereditary cancer [6] (Table 1).

Positive cases were defined as those having at least one likely pathogenic or pathogenic variant (hereby collectively referred to as disease causing variants, DV), while VUS cases were defined as those with at least one VUS finding, but lacking any DV. Monoallelic carriers of a DV in *MUTYH* were not included in the mutation positive cohort. The mutation negative cohort also included cases with variants classified as likely benign or benign, as well as synonymous variants located outside the exon/intron junctions.

## Results

### Study population

**Fig. 1:**
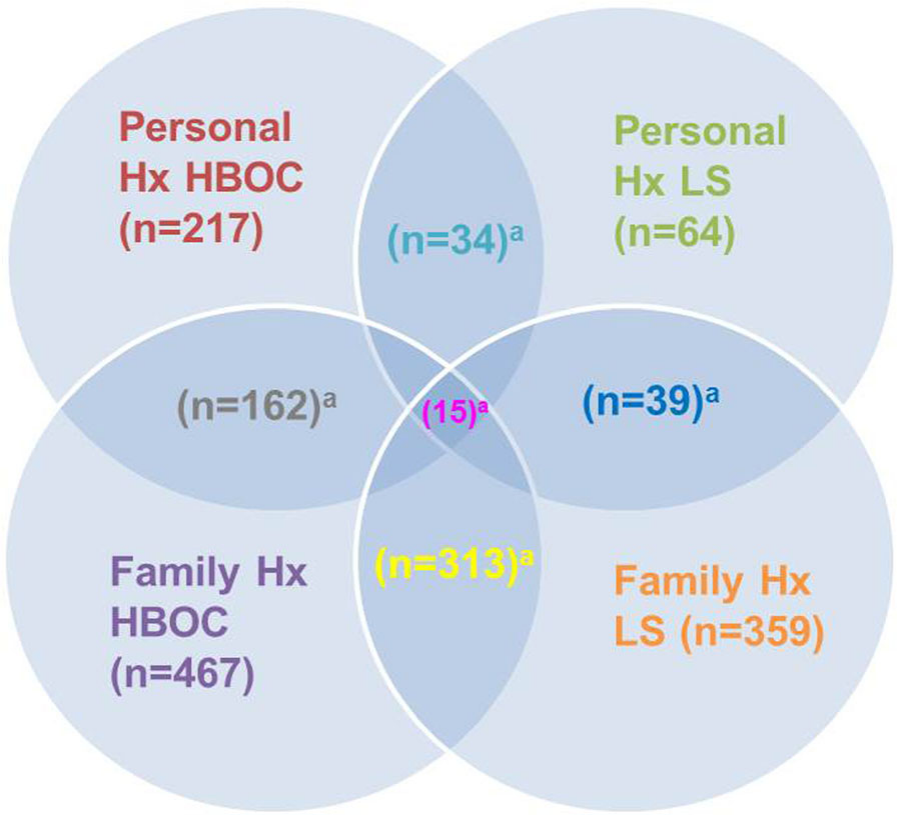
Clinical history for the 630 cases referred for VistaSeq Herditary Caner Panel Testing. ^a^The numbers provided in the figure represent an overlapping dataset that have not been removed from the four major phenotypic groups

Among the 630 cases referred for testing, 90% (*n* = 565) had an indication for testing provided by the referring physician. The median age of our cohort was 49 years old (range of 6–85 years old), with 94% female and 6% male. A family history of cancer constituted a major reason for referral, accounting for 84% (*n* = 529) of our cohort, while individuals with a personal history of cancer accounted for 44% (*n* = 276). Further evaluation of the cancer phenotypes revealed that 74% (*n* = 467) reported a family history of HBOC-associated cancers and 59% (*n* = 359) reported a family history of LS-associated cancers, with 50% (*n* = 313) reporting a family history of cancers associated with both syndromes (Fig. 1). A personal history of HBOC-associated cancers accounted for 34% (*n* = 217) of the cohort, while a personal history of LS-associated cancers accounted for 10% (*n* = 64). Five percent (*n* = 34) of the cohort reported a personal history of cancers associated with both HBOC and LS syndromes. Over half the patients (56%, *n* = 354) were unaffected and/or lacked clinical data.

### Variant and gene level distribution of DV

**Fig. 2:**
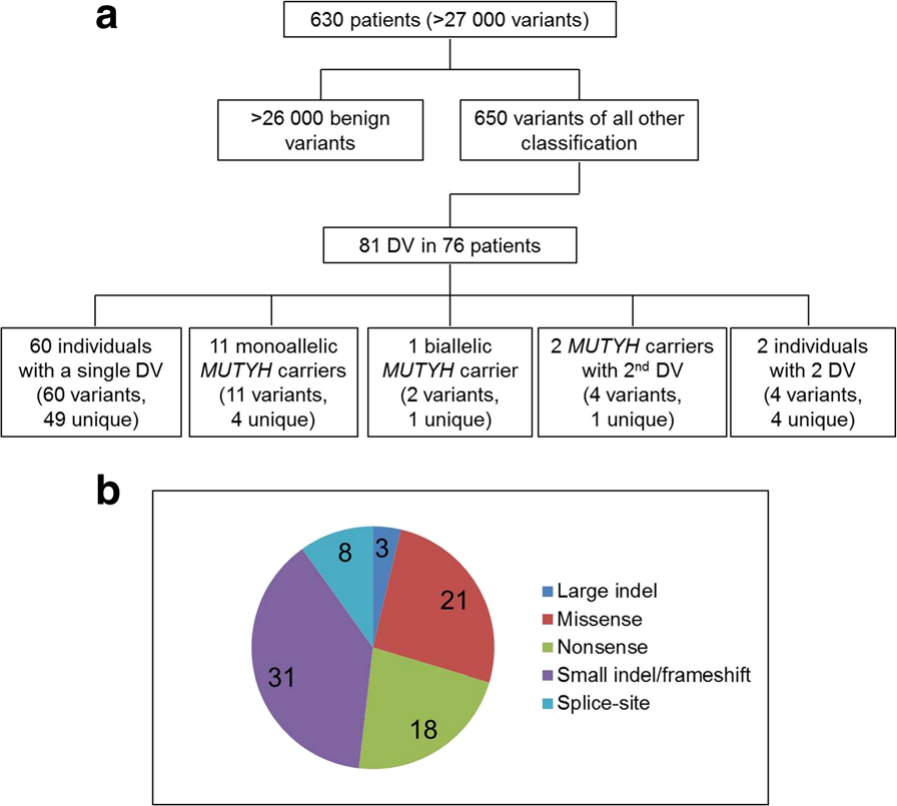
**a** Flowchart of variants detected in the cohort. **b**. DV by variant type. DV (*n* = 81) detected in 76 patients in the clinical cohort. Note: Non-unique variants, includes duplicates and monoallelic *MUTYH* variants

In the full dataset (*n* = 630 patients), over 27 000 variant occurrences were identified, of which 650 were classified within the spectrum of pathogenic to likely benign (Fig. 2a). Eighty-one of these were classified as DV and detected in 76 patients (12.1% of the study set; Additional file 1). Of those 76 patients, 60 carried a single DV in a gene other than *MUTYH*, 2 carried two DV in genes other than *MUTYH*, 11 carried a single DV in the *MUTYH* gene, and 2 had DV in both *MUTYH* and another gene. None of the *MUTYH*-only carriers had a personal history of LS. A homozygous DV in *MUTYH* was identified in one individual with a personal history of colonic polyposis and colorectal cancer. The *MUTYH* carrier frequency of 1/42 (2.4%) in our cohort was not significantly different from the frequencies of 1/49 (2.0%) [11], 1/38 (2.6%) [2], 1/37 (2.7%, clinical testing cohort) [7], 1/57 (1.75%) [10], and 1/56 (1.78%) [8] among 5 recently published reports (Chi-square test*, P > 0.05)*.

Seven DV were recurrent (detected in multiple individuals ranging from 2 to 9 in our dataset, Additional file 1), leaving a total of 59 unique DV in the dataset. 6.8% (*n* = 4) of these 59 unique DV were novel. The distribution of DV by variant type is represented in Fig. 2b. Frameshift and nonsense variants accounted for 60% (*n* = 49) of the DV spectrum, while missense, splice-site and large indels accounted for the remaining 26, 10 and 4% respectively. In all, 74% of the 81 DV were classified as pathogenic and 26% as likely pathogenic (Additional file 1).

Fifty-two out of the 59 unique DV were reported with classifications in the ClinVar database. These had an average of ~6 pathogenic/likely pathogenic classification submissions per variant. Fifty of the 52 variants (96%) reported in ClinVar had concordant classifications. The 7 variants that, to our knowledge, were not present in ClinVar include 3 previously reported large deletions (Additional file 1) and 4 novel variants (not reported in databases or literature).

DV were detected in 14 of the 27 genes included on the panel at the time of the study. The numbers of patients from each phenotypic group who carry variants in these genes are shown in Table 2. Forty-two percent (*n* = 34) of patients carried a DV in one of the classic HBOC- or LS-associated genes (*BRCA1/2*, *MLH1*, *MSH2*, *MSH6*, and *PMS2*), while the remaining 58% (*n* = 47) carried a DV in high risk genes for breast and other hereditary cancer syndromes (*n* = 17) or in genes associated with moderate to low risk (*n* = 30). The additional file included in this report provides the specific phenotypic data on the patient counts reported in Table 2.

**Table 2:** Spectrum of DV by gene, personal and family history of HBOC and LS

Gene Set	Gene	Total number of cases with DV	Individuals with Personal History^c^		Individuals with Family History^c^	
			HBOC	LS	HBOC	LS
BRCA	*BRCA1*	10	5	1	10	5
	*BRCA2* ^a^	10	3	0	8	6
Lynch	*MSH2*	4	1	2	3	4
	*MSH6* ^a^	3	0	1	0	2
	*PMS2* ^a^	7	1	0	4	3
High Risk	*CDH1*	1	0	0	1	1
	*APC*	1	0	0	0	1
	*MUTYH* ^b^	14	5	1	12	11
Moderate – Low Risk	*ATM*	8	5	0	5	7
	*BARD1* ^a^	1	0	0	0	0
	*BRIP1* ^a^	5	0	0	4	2
	*CHEK2* ^a^	9	3	3	7	3
	*NBN*	3	0	0	2	2
	*PALB2*	4	0	1	3	2

^a^No Clinical data was provided for 1 individual with DV detected in *BARD1, BRCA2, BRIP1, CHEK2, MSH6* and 2 individuals with a DV detected in *PMS2*

^b^One individual was biallelic for a DV in *MUTYH*, and two *MUTYH* DV carriers harbored a second DV in a different gene on the panel

^c^As ovarian cancer is a common clinical feature of both HBOC and LS, patients with ovarian cancer were included in both these categories

### Positive, VUS, and negative rates by phenotypic group

**Fig. 3:**
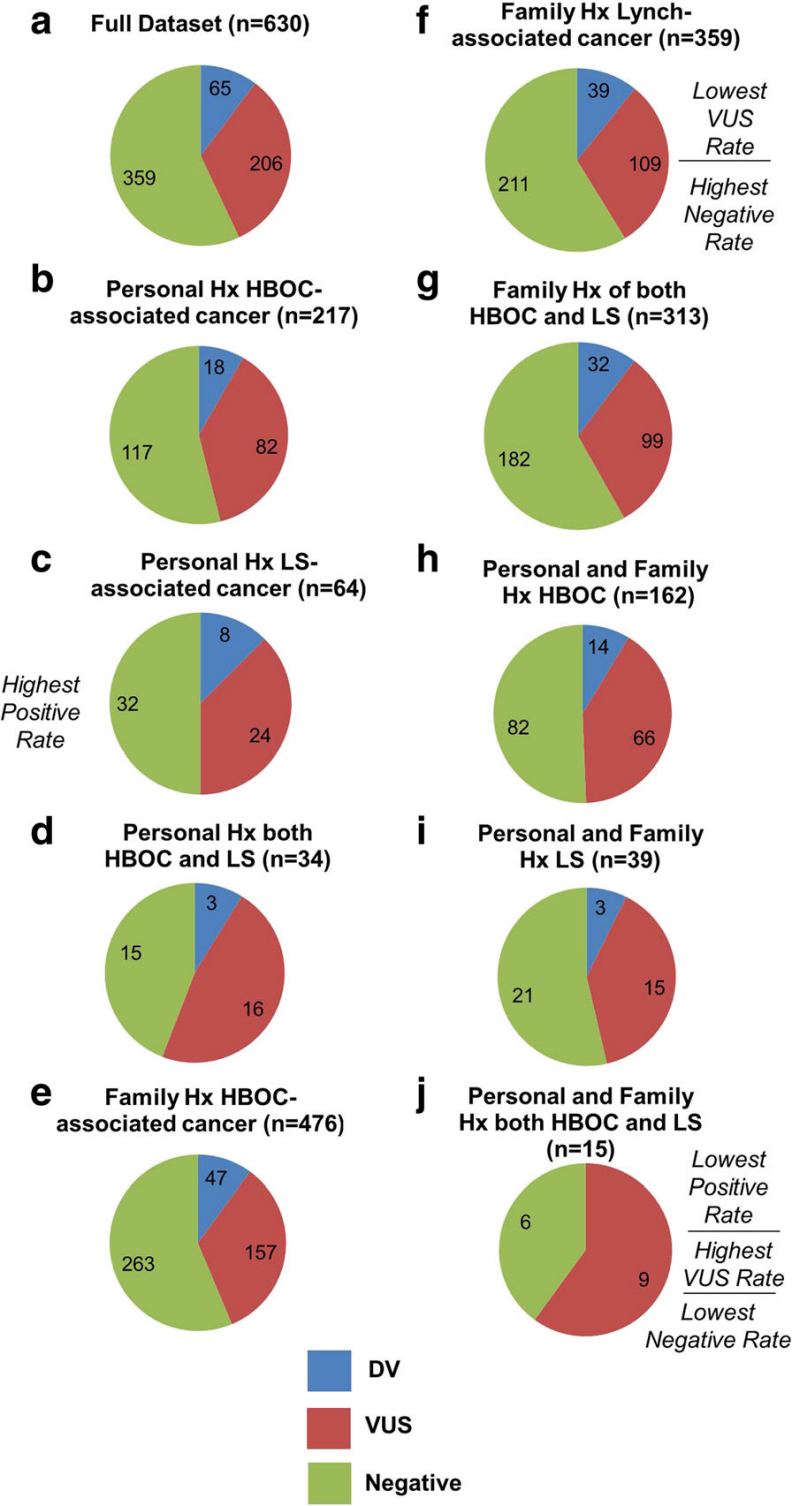
Percentage of positive, VUS, and negative result by phenotypic group

Excluding the carriers of DV in the *MUTYH* gene, a total of 68 DV were detected in 65 patients representing a 10.3% positive rate. Only 4.8% (n = 30) of patients in our cohort were tested based on a reported familial DV previously identified in one of the genes on our panel. Eleven of these patients tested positive for the familial DV, while one tested positive for a different DV. Even after excluding patients with a family history of a known variant, the positive rate remained at 9% (*n* = 53 out of 600 patients). This illustrates that our positive rate is not artificially inflated by inclusion of patients at high risk of carrying a known familial variant (Chi-square test*, P > 0.05)*, but reflects the actual percentage of family or personal history of cancer that can be attributed to DV in one or more of the genes on our panel. The overall VUS rate among the 630 cases in our dataset was 32.7% (*n* = 206). Eighty-one percent of these carried a single VUS, while 19% had more than one VUS. VUS were most commonly detected in *ATM* (*N* = 41), *APC* (*N* = 24) and *BRCA2* (*N* = 23); these three genes have the largest total coding region and could therefore be susceptible to more variation. The small subset of individuals with a personal and family history of both HBOC- and LS-associated cancers (*n* = 15) had the lowest positive rate (0%), highest VUS rate (60%), and the lowest negative rate (40%). The group with a personal history of LS-associated cancers had the highest positive rate (12.5%). The group with a family history of LS-associated cancers had the lowest VUS rate (30.4%) and the highest negative rate (58.8%). All other phenotypic groups had positive rates ranging from 7.7–10.9%, VUS rates ranging from 31.6–47.1%, and negative rates ranging from 44.1–58.1% (Fig. 3).

Four patients carried multiple DV, including two carrying a single *MUTYH* DV in addition to a DV in a different gene (*ATM* and *PMS2*). Of the two individuals who carried two DV in genes other than *MUTYH*, one was affected with colorectal cancer and carried a DV in *CHEK2* and *PALB2*, while the second was not personally affected with cancer but did have a family history of breast and gastric cancer and carried DV in *PMS2* and *CDH1* genes.

### Positive and VUS rates by gene panel

**Fig. 4:**
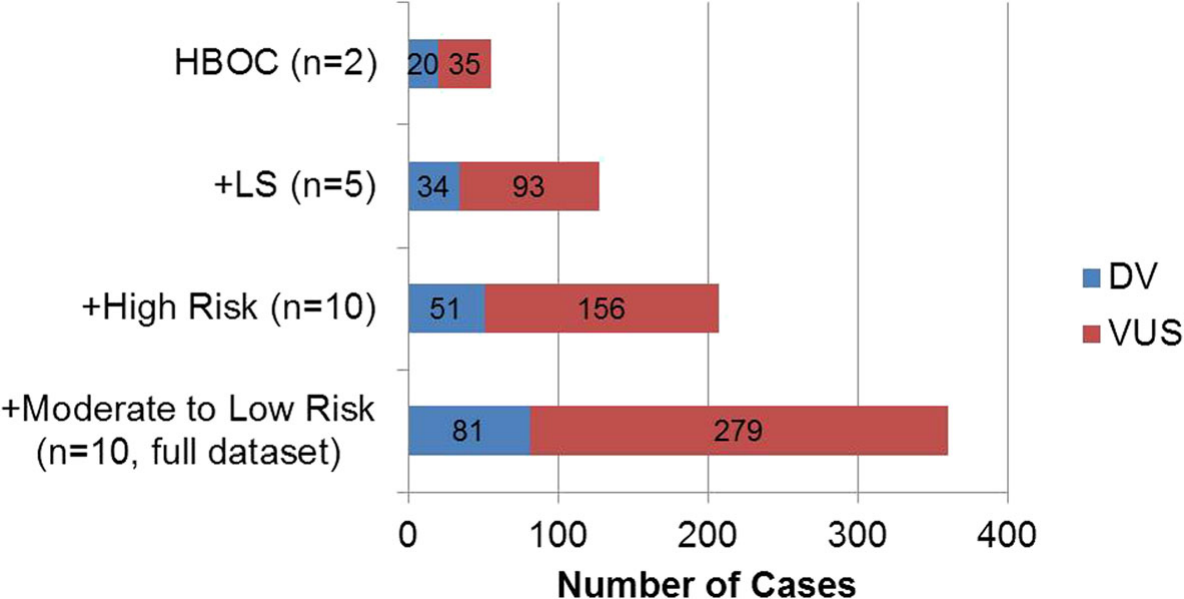
Positive and VUS rates with sequential inclusion of additional genes on the panel

The overall ratio of VUS: DV was lowest for BRCA genes (*n* = 2) at a ratio of 1.75:1, which continued to increase as more genes were added to the panel (Fig. 4). The sequential inclusion of LS genes (*n* = 5) yields a VUS: DV ratio of 2.7:1, increasing further to 3.1:1 with the inclusion of high risk genes (*n* = 10). Finally, inclusion of the remaining moderate and low-risk genes (*n* = 10) leading up to the complete 27-gene panel resulted in a VUS: DV ratio of 3.4:1. These results point to a significant increase in the VUS rate accompanying the proportional increase in positive rate with the inclusion of additional genes.

## Discussion

Table 3 summarizes our panel positive rate in relation to other recently published reports and illustrates the following trends. 1. The overall positive rate across similarly sized gene panels (27+/−2 genes), including the current study, is relatively uniform between 8 and 13% (median = 10.3%). 2. As expected, panels with fewer genes that include *BRCA1/2* tend to have a higher positive rate and a lower VUS rate presumably due to a narrower range of indications guiding test uptake. 3. Cohorts enriched with patients fulfilling either the NCCN guidelines for HBOC [7], LS [2] or society of gynecological oncology criteria for endometrial cancer [9] display lower positive rates for “non-BRCA” and “non-LS genes” relative to the *BRCA1/2* and LS genes.

**Table 3:** Comparison between the most recent reports on the performance of inherited cancer panels

Study	Sample Size	Panel Size	Panel Positive Rate^a^	VUS Rate	Genes in common	Panel Non-BRCA positive rate^a^	Panel Non-LS positive rate^a^	Study Cohort
Current Study	630	27 genes	10.3%	32.7%	–	7.1%	8.1%	Prospective commercial laboratory cohort unselected for NCCN, or previous BRCA/LS testing tested on a panel that includes *BRCA1/2* and LS genes
Lincoln, et al. 2015^7^	735	29 genes	12.4%^b^	41.0%^c^	23	3.5%	11.3%	NCCN HBOC enriched clinical referral cohort tested on a panel that includes *BRCA1/2* and LS genes
Yurgelun, et al. 2015^2^	1260	25 genes	12.4%	38.0%	25	11.2%	3.3%	NCCN LS enriched cohort tested on a panel that includes *BRCA1/2* and LS genes
Yurgelun et al. 2017^8^	1058	25 genes	8.2%	31.2%	25	7.2%	5.1%	Unselected CRC care clinic based cohort tested on a panel that includes *BRCA1/2* and LS genes
Ring, et al. 2016^9^	381	25 genes	9.2%	26.0%	25	8.7%	3.4%	Society of gynecologic oncology criteria for endometrial cancer enriched cohort tested on a panel that includes *BRCA1/2* and LS genes
Minion et al., 2015^10^	911	21 genes	7.4%	27.1%	19	7.4%	5.9%	BRCA1/2 test negative cohort with a personal history of HBOC tested on a panel that includes *BRCA1/2* and LS genes
Tung, et al. 2015^11,d^	1781	25 genes	13.5%	41.7%	25	4.3%	13.1%	Commercial laboratory *BRCA1/2* referral cohort tested on a panel that includes *BRCA1/2* and LS genes
LaDuca, et al. 2014^12^	425	22 genes	9.6%	23.5%	20	9.6%	7.8%	Commercial laboratory cohort tested on a cancer panel that did not include *BRCA1/2* but included LS genes
LaDuca, et al. 2014^12^	874	14 genes	7.4%	19.8%	12	7.4%	7.4%	Commercial laboratory high risk of HBOC selected cohort tested on a panel that did not include *BRCA1/2* but included LS genes
Mannan, et al. 2016^13^	141	13 genes	36.2%	14.8%	13	9.9%	36.2%	Unrelated patients and families with HBOC tested on a panel that did not include LS genes but included *BRCA1/2*
Susswein, et al. 2016^14,e^	2056	29 genes	10.2%	34.7%	25	Not inferred	Not inferred	Commercial laboratory referral cohort tested on a panel that includes *BRCA1/2* and LS genes

^a^All positive rates are calculated without including *MUTYH* carriers

^b^Panel positive rate was calculated from Supplemental Table S9, Clinical Referral Cohort (*n* = 735)

^c^VUS rate calculated from entire cohort, including Clinical Referral (n = 735), History enriched (*n* = 327)

^d^Positive and VUS rate calculated from cohort 1

^e^Only 2056 patients were tested for the comprehensive 29 gene panel

Recent studies that were enriched for patients fulfilling the NCCN criteria for HBOC [7] and LS [2] reported identical overall panel positive rates of 12.4% (Table 3) with *BRCA1/2* and LS genes contributing the major source of positive cases (*BRCA1/2*, 66/735, clinical referral cohort and LS, 114/1260 at 9.0% each). As would be expected, the corresponding non-*BRCA* (26/735) and non-LS (42/1260) positive rates in these studies were lower at 3.5 and 3.3%, respectively. In further support of this observation, a similar trend of a lower non-LS positive rate was observed in another study reporting a selected cohort enriched of patients with endometrial cancer [9], which is presumably similar to the NCCN LS-enriched cohort in its characteristics (Table 3). A recent follow-up study has demonstrated an increase in non-LS positive rate to 5.1% when testing was performed on a “NCCN unselected” colorectal cancer clinic based cohort [8]. As expected, our study, which represents a clinical laboratory referral cohort not enriched for patients preselected to fulfill the NCCN guidelines, yields a proportionately higher positive rate for non-BRCA 7.1% (45/630) and non-LS 8.1% (51/630) genes while yielding a lower positive rate of 3.2% for *BRCA1/2* (20/630) and 2.2% for LS (14/630) genes, respectively. A similar positive rate of 7.4% (67/911) for non-BRCA genes has been recently reported in a cohort of 911 subjects selected on the basis of a personal history of breast and/or ovarian, fallopian, or primary peritoneal cancer, who were previously tested to be negative for *BRCA1/2* genes [10]. This cohort was also not selected for enrichment based on NCCN guidelines. Several factors influence the test performance reported in each of these studies, such as the type of cohort, if enriched for family history or not, the number and content of genes on a panel, and differences in approaches to variant classification. Out of the 81 DV identified in this study, 42% were detected in either of *BRCA1/2* or LS genes, while 58% were detected in other genes on the panel, demonstrating a higher positive rate for non-BRCA and non-LS genes in our study relative to cohorts enriched for NCCN criteria.

We did not observe a significant enrichment for carriers of *MUTYH* in our referral cohort relative to the estimated frequency of 1–2% reported in the general population [4]. Furthermore, the estimated carrier frequency of *MUTYH* in our clinical referral cohort (1/42, 2.4%) is consistent with several recently published reports enriched for patients based on NCCN guidelines for breast and colorectal cancer. The risk for developing colorectal cancer among carriers of *MUTYH* is unclear with some studies reporting an increase in colorectal cancer risk among carriers with an affected first-degree relative, [23–25] while others have found no significant increase in the risk of colorectal cancer [26]. The lack of significant differences in *MUTYH* carrier frequency across multiple independent studies, including this report, is consistent with current NCCN guidelines that suggest tailored surveillance based on individual and family risk while not proposing specific recommendations for medical management among *MUTYH* carriers [27].

Out of the 23 patients with a personal history of HBOC-associated cancer who were found to carry a DV, only 35% (*n* = 8) had a DV in *BRCA1/2*, while the majority of these positive cases had a DV in non-BRCA genes. Similarly, out of the 8 patients with a personal history of LS-associated cancers who were found to carry a DV, only 38% (*n* = 3) were in LS-associated genes. The same findings hold true when considering family history. Out of the 56 DV-positive individuals with a family history of HBOC-associated cancers, only 32% (*n* = 18) were found to have DV in *BRCA1/2* and of the 47 DV-positive individuals with a family history of LS-associated cancer, only 21% (*n* = 10) carry DV in LS genes. These results emphasize the clinical utility of larger gene panels, as the majority of positive cases would have been missed, if they would have been tested solely for mutations in genes that were clinically indicated.

Interestingly, there were 15 individuals in the dataset who had both a personal and a family history of both HBOC- and LS-associated cancer, including 10 patients with ovarian cancer (which falls under both syndromes) and 5 individuals with both breast and colon cancer. Family history for these individuals was extensive and included at least two types of cancer in the family, and up to 7 types of cancer within a single family. However, despite a strong personal and family history of cancer, no DV was detected in this small subset. One possible explanation for this may be that these individuals carry DV in untested genes, further supporting the expansion of hereditary cancer gene panels, although the contribution of environmental factors leading to familial cancer susceptibility cannot be entirely excluded.

In the first 630 patients tested with the 27-gene panel, a DV was detected in 14 genes. After the collection of data for this study, additional DV have been detected in other genes including *BMPR1A*, *FAM175A*, *MLH1*, *RAD51C*, *RAD51D*, *SMAD4*, *STK11*, and *TP53*. Of note, although *MLH1* represents about 50% of all LS cases [4], no DV in this gene were detected via the 27-gene panel during the study period, and a limited number have been detected after the conclusion of the study. The reason for the underrepresentation of *MLH1* DV within this panel testing may be attributed to either a low percentage of individuals reporting a personal history of LS in our cohort (10%, *n* = 64) or an increased uptake of the specific LS gene panel prior to the consideration of expanded panels such as ours. In our experience, the vast majority of *MLH1* DV were detected by *MLH1* single gene sequencing (60%) or the broader 4 gene LS gene panel testing (35%), while only a handful of *MLH1* DV were detected through the 27-gene panel, providing support for this finding.

We observed a 4-fold increase in the number of DV detected when going from *BRCA1/2* genes upwards to the 25 additional genes on the panel. This increase in the positive rate clearly offsets the concomitant 8-fold increase in VUS. The positive rate as each gene set is added continued to increase, without a plateau being observed, suggesting that inclusion of additional genes to the panel offers room for a further increase in the overall positive rate. However, the critical number of genes before the positive rate plateaus towards the asymptote remains to be determined.

Three patients were found to have large deletions: one *APC* promoter deletion in a patient with an extensive family history of cancer, a multiple-exon deletion in *BRCA1* in a patient with personal and family history of HBOC-associated cancers, and a large multiple exon deletion in *PMS2* in a patient with a family history of both LS- and HBOC-associated cancer. Other studies testing for large deletion/duplication analysis within hereditary cancer panels have reported a range of positive findings from 0.7–2% [7, 14, 28] of the entire testing population, which is within range of 0.5% in our cohort.

Fifty out of the 52 DV in our dataset (96%) had concordant classifications in the ClinVar database. Four variants had ClinVar entries with other classifications in addition to pathogenic and likely pathogenic (see Additional file 1). *BRIP1* c.2392C > T (p.Arg798Ter) had a VUS and a pathogenic entry relative to phenotypes of breast cancer and Fanconi Anemia respectively, both contributed by the same submitter. As heterozygous carriers of truncating mutations in *BRIP1* have been reported to be susceptible to breast cancer [29], this VUS entry represents a pathogenic low penetrance classification that is currently not amenable to ClinVar submission requirements [30]. Similarly, *CHEK2* c.1555C > T (p.Arg519Ter) has a VUS entry without supporting evidence by a submitter, last evaluated in 2015. However, the corresponding publication by the same submitter cited in ClinVar did not list this variant among the reported VUS in *CHEK2*. Therefore, these two variants were not counted as discordant. The third variant, *ATM* c.4394 T > C (p.Leu1465Pro) has a VUS entry by a submitter, last evaluated in 2016, while acknowledging the experimental and clinical studies supporting a damaging role of the variant. This relates to differences originating from evidence weighting among submitters contributing to public databases. Lastly, *PMS2* c.2186_2187delTC (p.Leu729Glnfs) has one VUS entry and a likely benign entry, based on its high frequency in control databases (ExAC) and the possibility of this variant coming from *PMS2CL,* the *PMS2* pseudogene. We have observed this variant in several patients with either a personal and/or a family history of breast, brain, endometrial, cervical and uterine cancers referred for testing at our laboratory. In all our cases, we confirmed the presence of this variant in *PMS2* gene by long-range PCR. Furthermore, literature evidence describing this variant reports confirmation of this variant by long-range PCR and its segregation in individuals with features of Lynch and Constitutional Mismatch Repair Deficiency [31] (CMMRD) phenotypes. Recent studies have reported the presence of biallelic *PMS2* mutations in up to 60% of patients with CMMRD [31] in contrast to LS patients, the large majority of whom are carriers of *MLH1* and *MSH2* mutations. Furthermore, studies in unselected cohorts of colorectal cancer patients have demonstrated a higher prevalence of *PMS2*-associated LS than previously thought [32]. Nevertheless, its presence at a high frequency in ExAC could arise from a sequencing misalignment/pseudogene issues within the ExAC dataset, a higher carrier frequency for CMMRD reflecting the reduced penetrance of heterozygous *PMS2* mutations, or a combination of these factors.

This study was limited by the mode of data collection as the personal and family histories of cancer were ascertained from the self-reported information provided on requisition forms at test intake. Therefore, some of the information provided may not have been exhaustive for personal and family histories, including previous *BRCA1/2* or LS testing history and fulfillment of the NCCN guidelines as part of insurance coverage requirements prior to ordering genetic testing. Although these factors could be sources of potential bias in our cohort, they do not seem to inflate our tested *BRCA* and LS positive rates. The higher positive rates for non-*BRCA* and non-LS genes in our study relative to previous reports point to some unique features of our dataset. Additional studies would be needed to independently confirm these findings in unbiased datasets. For a subset of our cohort, no reasons for referral were provided. This includes seven individuals in whom a DV was identified. Another limitation is posed by the dynamic nature of variant classifications. All variants reported in this study represent a snapshot in time limited to the classifications obtained within the range of data collection. Concomitantly, the VUS rates reported are subject to some change as variants are re-evaluated at defined intervals. However, this is unlikely to have a significant impact on positive rates as most VUS move towards the normal spectrum as additional information becomes available. Lastly, although the overall sample size of our cohort was comparable to other published reports, the smaller subset of patients within each phenotypic grouping must also be considered as a potential limitation.

## Conclusion

The performance and detection rate of the 27-gene inherited cancer panel within a referral testing population correlated well with recently published reports. Despite an increase in VUS rate, additional DV continued to be detected with an increase in panel size, further corroborating the utility of panel testing in patients with an inherited predisposition to cancer. DV were detected in 14 out of the 27 genes during this study period although additional genes bearing actionable variants continue to be identified. DV in *BRCA1/2* were identified in patients with a personal/family history of LS and conversely, DV in LS-associated genes were identified in patients with a personal/family history of HBOC. This further supports consolidated panel testing over traditional criteria based genetic testing as the preferred assessment strategy to identify individuals with an inherited predisposition to cancer.

## Additional file

Additional file 1: Patient characteristics and variant listing among positive cases, Pathogenic and likely pathogenic variants detected in the study. (XLSX 20 kb)

## Abbreviations

BIC: Breast cancer information core
DV: Disease causing variant(s)
ExAC: Exome aggregation consortium
HBOC: Hereditary breast and ovarian cancer
LS: Lynch syndrome
NCCN: National comprehensive cancer network
VUS: Variants of uncertain clinical significance
